# Understanding the role of corporate social responsibility and sustainable supply chain management in shaping the consumers’ intention to use sharing platforms

**DOI:** 10.3389/fpsyg.2022.970444

**Published:** 2022-08-22

**Authors:** Wenjie Li, Idrees Waris, Chaojing Sun, Irfan Hameed, Muhammad Yaseen Bhutto, Rashid Ali

**Affiliations:** ^1^School of Business Administration, Shandong University of Finance and Economics, Jinan, China; ^2^Department of Management Sciences, University of Turbat, Turbat, Pakistan; ^3^Shandong Labor Vocational and Technical College, Jinan, China; ^4^College of Business Management, Institute of Business Management, Karachi, Pakistan; ^5^Faculty of Business and Management, UCSI University, Kuala Lumpur, Malaysia; ^6^Business School, Shandong Jianzhu University, Jinan, China; ^7^School of Information Engineering, Southwest University of Science and Technology, Mianyang, China; ^8^Department of Computer Science, University of Turbat, Turbat, Pakistan

**Keywords:** corporate social responsibility, eco-design, internal green management, green perceived quality, green concern, customer intention

## Abstract

Sustainable supply chain management (SSCM) in sharing economy platforms supports resource management and achieves environmental sustainability. Corporate social responsibility (CSR) is an essential pillar of sustainability, but the link between CSR and SSCM has been missing in the literature. Therefore, the current study intends to examine the connection between CSR and SSCM practices in sharing economy-based platforms. This study has applied the means-end theory to understand customer intention in the sharing economy. The data of 379 respondents from five main cities of Pakistan have been collected through convenience sampling. Partial least square structural equation modeling (PLS-SEM) has been used to test the proposed conceptual model. The study results show that the corporate social responsibility approach adopted by the sharing economy platforms improves internal supply chain management that drives customers’ intention to use sharing economy platforms. Green concern has a significant moderating effect on customers’ tendency toward environmental issues and solutions. However, findings revealed that eco-design in the sustainable supply chain does not affect customer purchase intention in sharing economy platforms. The study findings provide practical implications to organizations focusing on sustainable supply chain management practices in the sharing economy.

## Introduction

The influx of digital technologies has changed the ways of conventional businesses and opened avenues for new and sustainable businesses (Shahbaz et al., 2022). One of the developments in the sharing economy is a socioeconomic system built upon the sharing of physical, intangible services of human and intellectual resources. It includes the shared creation, production, distribution, trade, and consumption of products and services by different individuals and organizations (Makov et al., 2020). It is a business that rents and borrows products and services among peer-to-peer groups to maximize utilization (Hu et al., 2019). Furthermore, it promotes the maximum utilization of idle resources, environmental protection, and waste control (Dabbous and Tarhini, 2021). Organizations adopting sharing economy-based models do not own any products or assets but rely on digital technologies to trade and connect with people worldwide (Belk, 2014; Lee et al., 2018; Hu et al., 2019).

The business model of sharing economy has evolved significantly since 2010 with the advancement of key players in many sectors such as LendingClub (finance sector), Uber (automobile sector), Thredup (retail sector), Airbnb (hospitality sector), and Spotify (entertainment sector) through structural changes, technological developments and product developments (Hu et al., 2019; Gruber, 2020). The sharing economy explains the shared production, creation, distribution, and consumption of goods and services by different groups of people and organizations (Cheng et al., 2019). Participants of sharing economy depend on collaborative consumption by providing access to products and services owned individually. Boysen et al. (2019) define sharing economy as the collaborative consumption of goods and services by households and companies. Sharing economy-based companies strive to provide opportunities for different groups of people to access others’ resources (Malik and Wahaj, 2019). Sharing economy is a competitive business model that challenges traditional businesses due to its affordable services (Hu et al., 2019). It is a large-scale activity that maximizes profits and uses the resource.

The sharing economy idea is practiced in many of Pakistan’s business sectors, significantly benefiting businesses and consumers. Like other traditional businesses in Pakistan, sharing economy-based products and services do not acquire inputs, produce, or sell physical products. Instead, they invite participants (seller and service providers) and match them in different groups to access the other groups of participants (buyers and end-consumers). The sharing economy decreases inefficiency by making it easier to share resources on-demand. Alharthi et al. (2021) posited that the business model of sharing economy extends resource sharing to people to generate income. The sharing economy practice is not new to our society. It has been widely implemented in Pakistan in ride-sharing services such as Careem, Uber, and Bykea, and salon businesses such as Gharpar that provide home beautician services to male and female individuals.

Previous studies mainly focused on sharing economy in the context of tourism (Cheng et al., 2019), customers’ readiness to use ridesharing services (Wang et al., 2020), and the role of internet-based sharing in commercialized as well as non-commercialized settings (Weis, 2010). Researchers posited that the competitive advantage of the sharing economy could be explained through products and service quality, resulting in customer satisfaction and loyalty (Zhang et al., 2018). Similarly, Toni et al. (2018) explained that customer value is the most critical aspect of competitive advantage for a sustainable business in the sharing economy-based products and services. Extant literature has focused on different aspects of the sharing economy, such as accommodation, ridesharing, and clothing that attract customers’ attention. However, studies on social and economic practices of the sharing economy have not paid attention to the effectiveness of sustainable supply chain management practices on customer intention to use sharing economy-based products and services (Hu et al., 2019). SSCM incorporates green practices that fulfill the present generation’s needs without compromising future generations’ needs (Hu et al., 2019). Researchers suggested that sharing economy-based products/services lead to sustainability and build positive customer perceptions (Roos and Hahn, 2017). Scholars also argued that the promotion of capitalism has adversely affected environmental concerns in the sharing of economy-based products and services (Martin, 2016). In addition, many existing studies focused on the financial aspects of collaborative consumption services that benefit customers financially (Hamari et al., 2016; Liu and Mattila, 2017; Oyedele and Simpson, 2017). Due to these trends, businesses have not understood the relationship between the environment and customer perception toward sharing economy-based products and services (Thamsatitdej et al., 2017; Majumdar et al., 2021).

Applying SSCM practices in the sharing economy fulfills customer demand in a cost-effective and timely manner that finally satisfies the customers (Jermsittiparsert et al., 2019). SSCM integrates environmental and social goals that fulfill current generation requirements without compromising future resources (Hu et al., 2019). It incorporates essential pillars of environmental and social components of contemporary organizations. The present study conceptualizes five SSCM management practices: (1) CSR (social pillar), (2) IGM (environmental pillar), (3) ECD (environmental pillar), (4) GPQ (environmental pillar), and (5) GC (environmental pillar). The study by Ahmadi et al. (2017) highlighted the significance of the social pillar in achieving sustainability and better supply chain performances. In addition, SSCM helps procure sustainable products and effective reverse logistics that reduce environmental pollution (Hong et al., 2018; Muduli et al., 2020).

Few studies in the domain of sharing economy have paid attention to the environment as a pillar of sustainability and produced narrow findings. For example, some studies (Hamari et al., 2016; Hu et al., 2019) indicated that energy-saving, green management, eco-design, and green customer management are the main pillars of sustainability. However, they have ignored other important pillars of business sustainability, such as corporate social responsibility (CSR), green perceived quality, and green concern. Furthermore, previous studies lack the critical link between CSR and SSCM practices in the sharing economy-based platforms. The present study aims to fill the literature gap by assessing the nexus between CSR and SSCM practices (internal green management, green perceived quality, eco-design, green concern), driving customers’ intention to use sharing economy platforms. A more comprehensive model explains the effects of internal green management, green perceived quality, eco-design, and green concern on customers’ intention to use sharing economy platforms. This study empirically tested the research model on Uber, a popular sharing economy model. Uber is a ridesharing service that provides customers rent a ride service in Pakistan. It provides services as a broker that connects users and service providers and charges a commission for the rides.

The present study aims to understand customers’ intention to use sharing economy platforms based on SSCM practices. SSCM practices help the organization manage resources and improve environmental sustainability. Furthermore, the study analyzes the moderating effect of green concerns on the acceptance of sharing economy platforms.

**FIGURE 1:**
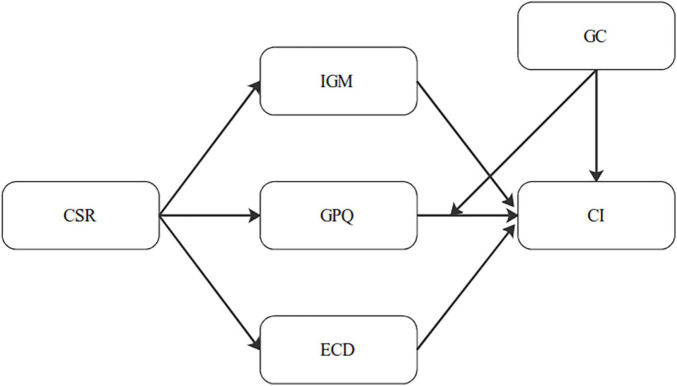
Conceptual framework. CSR, corporate social responsibility; IGM, internal green management; ECD, eco-design; GPQ, green perceived quality; GC, green concern; CI, customer intention.

The organization of the study is as follows: the first section is the introduction of the study that explains the importance of sharing economy and SSCM. The second section is the literature review and theoretical development. The third section of the article explains the methodology. The fourth section is the analysis. The last section is the discussions, implications, conclusion, and future research scope. Figure 1 is showing the conceptual framework of this study.

## Literature review and development of hypotheses

### Means-end chain theory

The means-end chain theory (MECT) suggests that consumers make a rational decision (Schaefers et al., 2021) and consume products and services that offer values at minimum utilization of resources (Costa et al., 2004; Hu et al., 2019). Customers use products and services that meet the required expectations and match their consumption values (Kang et al., 2020). From the perspective of sharing economy, researchers highlighted environmental (Hamari et al., 2016), financial (Guttentag, 2015), and social (Zhang et al., 2018) benefits to the customers. Sharing economy platforms have a vital role in achieving multiple goals: improving individual living standards, reducing resource production, and promoting environmental safety (Govindan et al., 2020). Therefore, researchers have highlighted the significance of environmental, social, and financial factors in increasing the adoption of sharing-based economy products and services (Hu et al., 2019).

The existing research on sharing economy is classified into two broad categories: organizational-level and individual-level. At the organizational level, research on sharing economy focused on model development and its application to industrial sectors (Binninger et al., 2015; Lee et al., 2018). At the individual level, research on sharing economy is limited. Few studies focused on factors affecting individual participation in the sharing economy. For example, the study by Hamari et al. (2016) indicated that financial incentives and enjoyment were significant predictors of individual participation in the sharing economy. Ballús-Armet et al. (2014) reveal that monetary saving, convenience, expanded mobility, and availability were significant factors of peer-to-peer ridesharing services. Extant literature on sharing economy is in its infancy because previous studies were mainly qualitative and conceptual, except for a few empirical studies (Möhlmann, 2015; Hamari et al., 2016; Hu et al., 2019). Hence, more empirical studies are required to study the factors affecting individual intention to use sharing economy platforms. Second, previous studies overlooked the link between CSR and SSCM driving intention to use sharing economy platforms. Practitioners’ aim should not only indicate the benefits of sharing economy-based products and services but also highlight the customers’ understanding and adoption of sharing economy-based products and services (Hu et al., 2019). Therefore, the current study establishes a conceptual framework based on the MECT to evaluate the link between CSR and SSCM practices adopted by sharing economy-based platforms and customers’ intention to use the sharing economy-based products and services.

### Corporate social responsibility

Corporate social responsibility encompasses business units’ philanthropic, moral, legal, and economic performances that extend to all stakeholders (Jones et al., 2017). Zhang et al. (2018) explained corporate social responsibility in organizations’ diversity management and participation in the local community. Liu and Lin (2020) posit that customers are more inclined to purchase manufacturers’ products that care about the sustainably of the environment. Customers’ sensitivity toward environmental issues affects manufacturers’ ethical behavior and contributes to the development of sustainable products (Khan et al., 2019). Organizations that emphasize sustainable supply chain management emphasize internal shareholders, channel partners, and external customers (Chuang et al., 2018). The researchers argued that firms that emphasize CSR would be more inclined toward green practices such as internal green management, eco-design, and green technology (Morea et al., 2021; Yang et al., 2021). Pino et al. (2016) indicated that CSR influences the producers’ legal responsibilities and ensures the availability of green products. Based on the previous extant literature, this study assumes that CSR activities of the sharing economy-based organizations lead toward SSCM. Hence, we propose the following hypotheses:

H1: Corporate social responsibility has a positive influence on internal green management of the sharing economy-based products and services.

H2: Corporate social responsibility has a positive influence on green product quality of the sharing economy-based products and services.

H3: Corporate social responsibility has a positive influence on the eco-designs of products of the sharing economy-based products and services.

### Internal green management

An organization’s green management practices denote the set of symbols, values (Wang et al., 2021), and internal green management that promotes effective employee–customer interaction (Hu et al., 2019). The firms’ internal measures help improve their environmental performance (Baah et al., 2021). Internal green management is a potential environmental pillar of sustainable supply chain practices (Zhang et al., 2018; Baah et al., 2021). Companies are practicing green management to attain dual benefits: to achieve profit, increase market share, and maintain the sustainability of the environment (Mojumder and Singh, 2021). Green management is gaining popularity because stakeholders are demanding environmentally friendly products and services that have a minimal adverse impact on environmental sustainability (Babiak and Trendafilova, 2011). Prior research shows that customers are more willing to pay for products and services from a business that considers environmental protection in their management practices (Hu et al., 2019; Mojumder and Singh, 2021). Therefore, we argue that internal green management practices in the sharing economy platforms would enhance green product quality. Hence, we propose the following hypothesis:

H4: Internal green management practices have a positive influence on customers’ intention to use sharing economy-based products and services.

### Green perceived quality

Quality of the products refers to consumers’ overall appraisal of the net benefit of a product (Zhao et al., 2021). Asgharian et al. (2012) posited that environmentalist trends and international regulations had urged companies to design green products to meet customers’ expectations of green products and promote environmental sustainability. Recently, green perceived quality has gained more significance due to its industrial and consumer purchase perspectives (Harju, 2022). The perceived quality of green products has dual effects: it maintains long-term relationships with the customers and affects their intention (Jaiswal and Kant, 2018). Customers’ intention increases if the perceived quality obtained from the green products is higher than that of the traditional competitive products (Majeed et al., 2022). Prior studies demonstrate that perceived quality positively influences customers’ intentions (Gil and Jacob, 2018; Wang et al., 2020). Based on green perceived quality literature, it can be assumed that green perceived quality obtained from the products and services of sharing economy affects customers’ intentions. Thus, we propose that the following hypothesis:

H5: Green perceived quality obtained in the SSCM positively influences customers’ intention to use sharing economy-based products and services.

### Eco-design

Eco-design incorporates environmental attributes into product development, thereby making it available to the designer to develop the product (Karlsson and Luttropp, 2006; Dahmani et al., 2021). In the beginning stage, companies implement eco-design by using white, gray, and black checklists for the products. Gray lists represent the use of materials based on good reasons. Blocklists contain illegal materials (Luttropp and Lagerstedt, 2006). Researchers highlight vital features that make up an eco-design: the integration of environmental attributes in product design and development process, the life cycle of green products at different stages, and its effects on the environment (Bovea and Pérez-Belis, 2012). Dangelico and Pujari (2010) highlighted the importance of eco-design in the product life cycle and argued that the market is unaware of the eco-design processes. Han et al. (2020) indicated that the eco-design of airport buildings positively affects the reputation of a company and drives customer purchases. However, Hu et al. (2019) found that eco-design practices adopted by sharing economy platforms do not drive customer intention. Therefore, it is essential to understand the impact of eco-design practices on customer purchase intention for sharing economy-based products and services. Hence, the following hypothesis is proposed:

H6: Eco-design practices have a positive influence on customers’ intention to use sharing economy-based products and services.

### Green concern as a moderator

Individual awareness regarding environmental issues and willingness to solve them represent green concerns (Zhang et al., 2018). Researchers attributed green concern as a direct and an indirect predictor of consumer intention (Newton et al., 2015; Mansoor and Paul, 2022), but very few studies considered the moderating effect of green concern (Zhang et al., 2018). Biswas and Roy (2015) posited that green consumers behave more environmentally friendly, such as participating in recycling and energy-saving behavior and purchasing environment-friendly products (Waris et al., 2021). Furthermore, Kwon et al. (2016) indicated that green concern is an effective moderator between third-party environment rating and brand greenness perception. In Pakistan, the prevailing sense of protecting the environment leads people to focus on protecting from natural hazards (Hameed et al., 2019). In line with this, customers with deep green concerns establish firm green beliefs in purchasing green products and services (Johnstone and Tan, 2015; Aslam et al., 2021). Hence, we argue that green concern moderates the relationship between green product quality and customer intention in the sharing economy-based products and services.

H7: Green concern has a positive impact on customer intention to use sharing economy-based products and services.

H8: The influence of green perceived quality of customer intention to use sharing economy-based products and services is moderated by green concern. The higher the green concern of the customer, the more positive impact green perceived quality will exert on customer intention to use sharing economy-based products and services.

## Methodology

### Data collection and sampling

The current study employed a convenience sampling technique for data collection. It is used to generate samples as per ease of access and readiness to be a part of the sample from the respondents. By using this technique, we observed the opinions of the customers of sharing economy regarding green practices performed by sharing economy platforms. The advantage of this type of sampling is that it is easy to access the data. The face-to-face self-administered data collection technique was used to understand customer intention. The data were mainly gathered from customers of sharing economy-based services in the cities of Karachi, Lahore, Sukkur, Faisalabad, and Islamabad. The reason for selecting these cities is that sharing services are available (Careem, Uber, and Bykea). The adequate sample size to conduct this research was 270, as suggested by Yang et al. (2017). However, to increase reliability, we have doubled this sample size to 620. A group of 15 MPhil students was hired for the distribution of the questionnaire; three students were selected to visit each city and collect data from respondents. They visited the cities where the concept of sharing economy exists and distributed the survey questionnaires. Finally, valid data of 379 respondents with a response rate of 61.12% were gathered. The rest of the questionnaires was either partially filled or had missing values.

### Instrumentation

A survey questionnaire was adapted from different sources and redesigned for data collection. The adapted items were modified by five marketing and supply chain experts. The questionnaire contains six variables and a total of 27 items. All the items were scaled on a five-point Likert scale, ranging from strongly disagree to strongly agree. The questionnaire was pre-tested to evaluate its reliability and validity. For the pilot study, 35 random respondents were selected to fill the questionnaire. The reliability of data collected from these respondents was checked. The respondents reported some ambiguities regarding the items of customers’ intention: “sharing economy-based products/services” that were later modified after consultation with the area experts. The modified questionnaire included “sharing economy-based services” only. For example, item 1: “I am willing to use sharing economy-based services in future.” The modified questionnaire was again presented to another 30 respondents. After achieving positive comments regarding the appropriateness of the questionnaire, it was then formally distributed to the target respondents. The sources of the measuring items are presented in Table 1.

**TABLE 1:** Measurements.

Variables	No. of items	References
Corporate social responsibility	4	Maignan and Ferrell, 2000; Turker, 2009
Eco-design	4	Fernando, 2017
Internal green management	5	Agyabeng-Mensah et al., 2020
Green perceived quality	5	Chen et al., 2015
Green concern	4	Zhang et al., 2018
Customer’s intention	5	Hamari et al., 2016

## Data analysis

Statistical Package for Social Sciences (SPSS) and partial least square structural equation modeling (PLS-SEM) have been used to analyze the collected data. SPSS has been used for data purification and assessing common method bias. However, PLS-SEM has been used to analyze measurement and structural models.

### Common method variance

Common method bias (CMB) may occur when a single source represents more than half of the variance caused by all factors (Podsakoff et al., 2003). The chances of CMB increase when there is a single source of data collection in a self-administered questionnaire. The anonymous usage questionnaire is one of the methods to overcome this issue (Miller and Cardinal, 1994). The variance explained by a single factor has been assessed using Harman’s single-factor test to assess the presence of CMB. It has been substantially identified that a single factor is causing only 27.625% of the variance.

Hence, according to the recommendation of Podsakoff et al. (2003), it has been inferred that the data are free from the issue of CMB.

### Profile of the participants

The data were collected from varying cities of Pakistan covering nearly all segments. Table 2 represents the demographic profile of the respondents. The majority of the respondents were men, accounting for 57.8% of the total responses. The representation of the respondents in terms of age was almost equally scattered toward all age groups; however, the people with the age range of 31–35 years were the highest (27.2) in number. Most (34%) of the respondents had the practice of using sharing economy-based services three to four times a week. In terms of income, 66.4% of the responses were from people with a monthly household income of 50,000 PKR or less, with 33.2% having income less than 25,000 PKR.

**TABLE 2:** Respondents’ profile.

	Demographics	Frequency	Percent
Gender	Men	219	57.8
	Women	160	42.2
Age	16–20	62	16.4
	21–25	86	22.7
	26–30	82	21.6
	31–35	103	27.2
	36 and above	46	12.1
Monthly frequency of using sharing economy services	1 to 2 times	122	32.2
	3 to 4 times	129	34.0
	5 to 6 times	85	22.4
	7 or more times	43	11.3
Monthly income	Less than 25,000	126	33.2
	25,000–50,000	126	33.2
	51,000–75,000	86	22.7
	76,000 and above	41	10.8

### Reliability and convergent validity

Data quality was assessed by measuring internal consistency, which was first measured through Cronbach’s alpha values. All of the values adhered to the threshold value (≥0.70). Further following the recommendation of Hair et al. (2016), the composite reliability (CR) technique has been used to assess the internal consistency of the data. Hair et al. (2016) further suggested that the CR is the better method for calculating internal consistency; all values were found within the acceptable range of 0.70. The correlation of the single construct with other constructs has been measured using convergent validity. The convergent validity is assessed by the average variance extracted and values of the outer loadings. Values of both analyses are within the acceptable range, with AVEs of all constructs above 0.50 and CR values above 0.70. Hence, the data meet the criteria of convergent validity (Hair et al., 2016), as shown in Table 2.

### Discriminant validity

According to Hair et al. (2017), the discriminant validity evaluates the extent to which a construct is unrelated to another construct in the study. Triangulation has been applied to calculate discriminant validity by smearing criteria, heterotrait-to-monotrait (HTMT) ratio, and cross-loading values. Fornell and Larcker’s (1981), Table 3 criterion that the square of AVE values must be greater than the corresponding correlations has been confirmed, as shown in Table 4. The construct values of all constructs are below 0.85, following the HTMT ratio standards (Henseler et al., 2015), as shown in Table 5. Discriminant validity has also been confirmed by cross-loading criteria, which state that each construct item must have higher cross-loading values than other constructs (Hair et al., 2017), as shown in Table 6.

**TABLE 3:** Reliability testing and convergent validity.

Constructs	Items	Loading	Cronbach’s alpha	CR	AVE
Customer intention	CI1	0.842	0.912	0.935	0.741
	CI2	0.792			
	CI3	0.907			
	CI4	0.910			
	CI5	0.848			
Corporate social responsibility	CSR1	0.795	0.870	0.911	0.720
	CSR2	0.869			
	CSR3	0.883			
	CSR4	0.844			
Eco-design	ECD1	0.871	0.904	0.934	0.782
	ECD2	0.967			
	ECD3	0.911			
	ECD4	0.776			
Green concern	GC1	0.717	0.716	0.825	0.542
	GC2	0.793			
	GC3	0.775			
	GC4	0.653			
Green perceived quality	GPQ1	0.666	0.804	0.864	0.562
	GPQ2	0.652			
	GPQ3	0.830			
	GPQ4	0.817			
	GPQ5	0.767			
Internal green management	IGM1	0.836	0.853	0.896	0.634
	IGM2	0.774			
	IGM3	0.880			
	IGM4	0.654			
	IGM5	0.818			

CR, composite reliability; AVE, average variance extracted.

**TABLE 4:** Discriminant validity analysis.

Latent variables	1	2	3	4	5	6
Consumer intention	**0.861**					
Corporate social responsibility	0.572	**0.848**				
Eco-design	0.219	0.234	**0.884**			
Green concern	0.435	0.335	0.222	**0.736**		
Green perceived quality	0.558	0.463	0.103	0.370	**0.750**	
Internal green management	0.366	0.311	0.575	0.357	0.234	**0.796**

The bold diagonal values refer to the square root of the AVE of each construct. All correlations are statistically significant (*p* < 0.01).

**TABLE 5:** Heterotrait-to-monotrait (HTMT) ratio results.

Latent variables	1	2	3	4	5
Consumer intention					
Corporate social responsibility	0.642				
Eco-design	0.234	0.258			
Green concern	0.535	0.419	0.271		
Green perceived quality	0.635	0.537	0.117	0.492	
Internal green management	0.403	0.347	0.654	0.446	0.282

**TABLE 6:** Cross loadings.

	CI	CSR	ECD	GC	GPQ	IGM
CI1	**0.842**	0.476	0.231	0.329	0.466	0.406
CI2	**0.792**	0.460	0.108	0.306	0.449	0.229
CI3	**0.907**	0.562	0.206	0.406	0.493	0.355
CI4	**0.910**	0.517	0.235	0.443	0.539	0.331
CI5	**0.848**	0.441	0.146	0.376	0.445	0.237
CSR1	0.539	**0.795**	0.218	0.264	0.358	0.291
CSR2	0.456	**0.869**	0.233	0.318	0.425	0.312
CSR3	0.484	**0.883**	0.191	0.272	0.384	0.223
CSR4	0.464	**0.844**	0.144	0.276	0.398	0.219
ECD1	0.197	0.178	**0.871**	0.182	0.104	0.509
ECD2	0.239	0.250	**0.967**	0.239	0.127	0.583
ECD3	0.168	0.183	**0.911**	0.199	0.057	0.500
ECD4	0.158	0.206	**0.776**	0.156	0.064	0.422
GC1	0.301	0.197	0.207	**0.717**	0.203	0.309
GC2	0.340	0.331	0.228	**0.793**	0.280	0.369
GC3	0.341	0.249	0.146	**0.775**	0.277	0.196
GC4	0.297	0.200	0.068	**0.653**	0.332	0.172
GPQ1	0.299	0.284	0.064	0.308	**0.666**	0.124
GPQ2	0.473	0.410	0.094	0.247	**0.652**	0.231
GPQ3	0.461	0.411	0.086	0.292	**0.830**	0.209
GPQ4	0.416	0.312	0.056	0.305	**0.817**	0.101
GPQ5	0.391	0.268	0.077	0.241	**0.767**	0.185
IGM1	0.323	0.288	0.549	0.312	0.239	**0.836**
IGM2	0.220	0.161	0.502	0.226	0.180	**0.774**
IGM3	0.344	0.302	0.445	0.367	0.260	**0.880**
IGM4	0.256	0.250	0.391	0.170	0.036	**0.654**
IGM5	0.282	0.197	0.403	0.309	0.187	0.818

The bold diagonal values refer to the square root of the AVE of each construct. If the diagonal values in the same column is greater than the other values it means discriminant validity is established.

### Predictive power of the inner model

The inner model fitness has been assessed using the coefficient of determination (R2) and predictive relevance through the value of cross-validated redundancy (Q2). The R2 value is the percentage of the effect of predicting variables on the outcome variables. The R2 value of 39.7% represents moderate to high predictive accuracy. The cross-validated redundancy (Q2) was checked using the blindfolding method. The predictive relevance in the model is confirmed when the value of Q2 is greater than 0. The Q2 value of 29.1% of the proposed model is considered as substantial predictive relevance (Henseler et al., 2015).

Table 7 shows the results of hypothesis testing under the p-value and t value criteria. As mentioned in Table 7, corporate social responsibility has a significant and positive influence on internal green management, and eco-design of green products refers to the acceptance of H1, H2, and H3, respectively. Internal green management and green perceived quality positively and significantly affect customer intention referring to the acceptance of H4 and H5. However, the positive influence of eco-design on customer intention was insignificant. Thus, H6 was rejected. The positive and significant effect of green concerns on customer intention was also confirmed, which refers to the acceptance of H7. Green perceived quality and green concern interaction have a positive and significant influence on customer intention, which refers to the acceptance of H8.

**TABLE 7:** Hypotheses testing.

Hypotheses	Beta	*p*-values	*t*-values	Decision
H1: CSR ->IGM	0.311	0.000	5.787	Supported
H2: CSR ->GPQ	0.463	0.000	11.236	Supported
H3: CSR ->ECD	0.234	0.000	4.117	Supported
H4: IGM->CI	0.174	0.003	2.961	Supported
H5: GPQ ->CI	0.438	0.000	10.190	Supported
H6: ECD ->CI	0.029	0.569	0.569	Not supported
H7: GC ->CI	0.204	0.000	4.510	Supported
H8: GPQ*GC ->CI	0.098	0.000	4.013	Supported

Beta, standardized coefficient path; SE, standard error. Significant at (*p* < 0.05).

### The results of moderating effect

**FIGURE 2:**
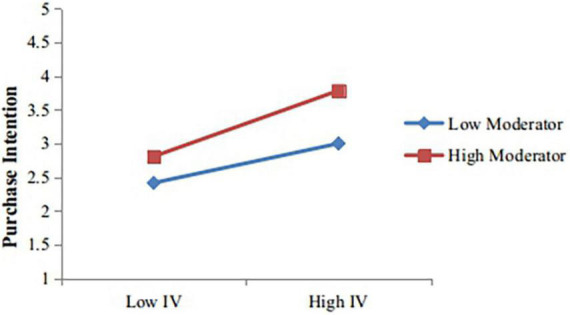
Moderating effect of green concern.

We have performed two procedures to test the moderating effect of green concerns. The first step was performed to avoid the equal contribution of the variables from different measurement scales. For this purpose, the independent and moderating variables were standardized to create interaction terms for both. Second, we placed the dependent variable into the equation, and then the independent variable and interaction variable were placed in the sequence. Table 7 shows that green concern has a positive and significant influence on customer intention (β = 0.270, *p* < 0.000). Then we introduced the interaction term of standardized independent and moderating variables. Table 7 shows that green concern moderates the relationship between green product quality and customer intention (β = 0.098, *p* < 0.000). To indicate the moderating effect of green concern, the relationships were replotted at the two levels (high level and low level) (Li and Tang, 2010). The moderating effect of green concern is shown in Figure 2. At the low level of green concern, customer intention increases from 2.414 to 2.998. At the high level of green concern, customer intention increases from 2.806 to 3.782. This signifies that at a higher level of green concern, the strength of the relationship between perceived green product quality and customer intention is high.

## Discussions

This study is based on the means-end chain theory in the sharing economy economy-based services to predict customer intention to use sharing economy products and services. The role of CSR is essential in improving the local community and contributes to the betterment of society. For example, sharing economy progress will generate millions of job opportunities that improve the living standard of the communities. Previous studies extensively observed consumer intention in the sharing economy-based products and services (Yang et al., 2019; Ek Styvén and Mariani, 2020). However, studies failed to establish a link between CSR and SSCM. Therefore, the current study intends to examine the connection between CSR and SSCM practices in the sharing economy-based platforms. SSCM has a crucial impact on consumer decision-making regarding purchasing environmentally friendly products and services. The study results depict that CSR is essential in developing internal green management practices, green perceived quality, and eco-design of the products. These findings are consistent with prior studies where researchers argued that CSR has a significant influence on the internal green management of the activity of the firms (Chuang et al., 2018; Anser et al., 2020). The positive influence of CSR on the eco-design of the products is also consistent with prior studies where researchers found that CSR activities of the firms affect eco-design (Yu et al., 2008; Morea et al., 2021). Furthermore, the positive influence of internal green management on customer intention is consistent with prior studies (Hu et al., 2019; Mojumder and Singh, 2021). These findings are consistent with previous studies that signify the role of CSR in the sharing economy products/services (Martinez and Bosque, 2013; Hu et al., 2019). The positive effect of CSR on SSCM practices signifies that customers are attracted to sustainable practices of sharing economy platforms. The contribution of sharing economy platforms to local communities will enhance its image and increase customer loyalty.

The results signify that internal green management is an essential factor of the sustainable supply chain that affects customer intention to use sharing economy-based services. The study findings reveal that green perceived quality has a significant and positive influence on customer intention, which matches the results of previous studies (Jaiswal and Kant, 2018; Wang et al., 2020). However, the current study results are inconsistent with prior studies regarding the effectiveness of eco-design in driving customer intention (Park and Tahara, 2008; Delmas and Gergaud, 2021). IGM, ECD, GPQ, and GC are fundamental green-related practices of SSCM (Newton et al., 2015; Zhang et al., 2018). Hamari et al. (2016) posited that SSCM management practices significantly influence customers’ intentions. Consistent with the findings of Hamari et al. (2016), this study revealed that IGM, GPQ, and GC significantly influence customers’ intention to use sharing economy products/services. IGM, GPQ, and GC positively influence because these measures are easily noticeable to customers. However, the impact of ECD was non-significant and consistent with the findings of Hu et al. (2019). The insignificant impact is due to a lack of promotional activities on the sharing economy platforms. The lack of promotional activities regarding SSCM makes the customers less aware of sharing economy green management practices. Finally, to effectively communicate environmental practices of sharing economy platforms, focusing more on intrinsic attributes and green practices in the advertisement would be a better way to attract customers.

## Theoretical implications

The empirical findings of this study offer several theoretical implications. First, the study applied the SSCM concept and used novel constructs to predict customer intention to share economy-based products and services. It is among the first customer-centric studies that comprehensively focused on CSR and factors of SSCM in the sharing economy-based products and services. Second, the study provides valuable empirical insights that foster an understanding of SSCM factors and their effect on customer intention to use sharing economy-based products and services. Empirical evidence also helps understand the role of organization CSR activities on the elements of SSCM, which remains an issue of major concern for the organizations (Feng et al., 2017; Wang et al., 2020). Third, the study contributes to the means-end chain theory and proof that customers try to assess those products that meet sustainable supply chain processes and offer high-quality green products and services. Furthermore, despite CSR effectively influencing SSCM, the relationship between eco-design and green perceived quality was insignificant, which offers more grounds for empirical studies. Thus, the antecedents and consequences of the SSCM model in the sharing economy included CSR, internal green management, green perceived quality, and green concern. It was also found that green concern in sharing economy was an observed significant factor by the customers as it was also a significant moderator in the model. The findings of this empirical study could be contributed not only to sharing economy literature but also to the SSCM literature.

## Policy implications

This study provides practical implications from the sharing economy perspective and environmental sustainability. The sharing economy concept is gaining momentum, and it would be among business models due to resource constraints and environmental benefits. The model of SSCM depicts that customers are willing to use sharing economy-based products and services for resource conservation and environmental sustainability. The current study considered CSR activity a significant driver of organization business functions that help drive customer purchases. The effectiveness of CSR activities offers new insights to the business to adopt the model for sustainable business operations. Therefore, sharing economy platforms should focus on CSR activities to provide unique products and services that meet customers’ expectations. In addition, sharing economy platforms can work with local communities for the promotion of their culture and job creation to increase customer loyalty and financial performance. Moreover, CSR can enhance sharing economy’s internal performance by focusing on internal green management, green perceived quality, and eco-design of the products. Businesses in the sharing economy-based products and services should enhance internal green management practices, ensure green perceived quality, and design products that meet environmental standards. Researchers argued that the green perceived quality of the products increases the probability of the products’ purchase (Walia et al., 2020; Wang et al., 2020; Roh et al., 2022). Customer intention to use green products and services can also be increased by providing good value so that customers may get the value they perceived. Organizations must be certified by ISO14000 standards to enhance their visibility (Zhu and Cote, 2004). ISO 14000 certifications will benefit at the corporate level with excellent operation and improve the financial performance of the sharing economy platforms (Zhu and Cote, 2004; Centobelli et al., 2021). In addition, green practices should not be limited to the internal structure but include other supply chain actors to effectively establish SSCM practices in the sharing economy (Hu et al., 2019; Mallikarathna and Silva, 2019). Furthermore, the positive moderating effect of green concern implies that customers care about products and service quality when making decisions. Therefore, the companies need to provide authentic information related to green products of sharing economy that increase the acceptance of products and contribute to environmental sustainability.

## Conclusion and future research scope

Although this study covers a broader perspective of sharing economy, certain limitations can be addressed in future studies. First, the study has only focused on the customers’ perspective, while in sharing economy, other stakeholders also play an essential role, such as employees, suppliers, and investors. Therefore, it is recommended to include different stakeholders contributing to sustainability through sharing economy platforms. Furthermore, this study focuses on the service sector in sharing economy platforms in the country, and samples have been included from ride-sharing users only. Future studies may explore additional areas of sharing economy-based products and services and assess the customer behavioral intention. The discussion on the integration of CRS in the supply chain of sharing economy is limited compared with related sustainable supply chain management themes. Therefore, CSR should be emphasized in the sustainable supply chain management of sharing economy, including ethical working conditions and human rights. Most of the previous studies were conducted qualitatively and used conceptual models. There is a lack of quantitative study research. At present, only one study has systematically proposed the SSCM model under the sharing economy platform (Hu et al., 2019). Therefore, future studies can empirically analyze the impact of SSCM practices under the sharing economy platforms. The current study applied a quantitative approach to collect respondents’ primary data. Future studies can focus on groups of customers who are frequent users of sharing economy platforms. These results would provide a more comprehensive understanding of the phenomenon. In addition, the current study has not included the effects of gender. Future studies can evaluate the difference between male and female behavioral intentions to use sharing economy-based products and services. In the digital era, technology offers unprecedented opportunities to organizations for the management supply practices that contribute to environmental sustainability (Centobelli et al., 2020). Most of the sharing economy platforms work through a centralized supply chain due to which the personal data of the customers are at risk (Azzi et al., 2019). The implementation of blockchain technology eliminates intermediaries in the supply chain and prevents personal data fraud when individual nodes are attacked by hackers (Lim et al., 2021). Therefore, future studies should examine the impacts of blockchain technology in the supply chain of sharing economy and its impact on customers’ intention to use sharing platforms.

## Data availability statement

The original contributions presented in the study are included in the article/Supplementary material, further inquiries can be directed to the corresponding author.

## Ethics statement

This study has been reviewed and approved by the Directorate of Academics, University of Turbat, Pakistan. Written informed consent to participate in this study was provided by the participants’ legal guardian/next of kin.

## Author contributions

WL, IW, and CS conceptualized the topic, designed the methodology, and performed the data analysis. CS and IH helped in writing the first draft of the manuscript. WL, IW, CS, and MB worked on conclusion and implications. IW, IH, MB, and RA worked on the final draft of the manuscript. IW and RA edited the whole manuscript. All authors contributed to the article and approved the submitted version.
