# Supplementary figures and images for: Understanding the role of corporate social responsibility and sustainable supply chain management in shaping the consumers’ intention to use sharing platforms
Source: Front Psychol. 2022 Aug 22;13:970444. doi: 10.3389/fpsyg.2022.970444 (PMC9443847; doi:10.3389/fpsyg.2022.970444)

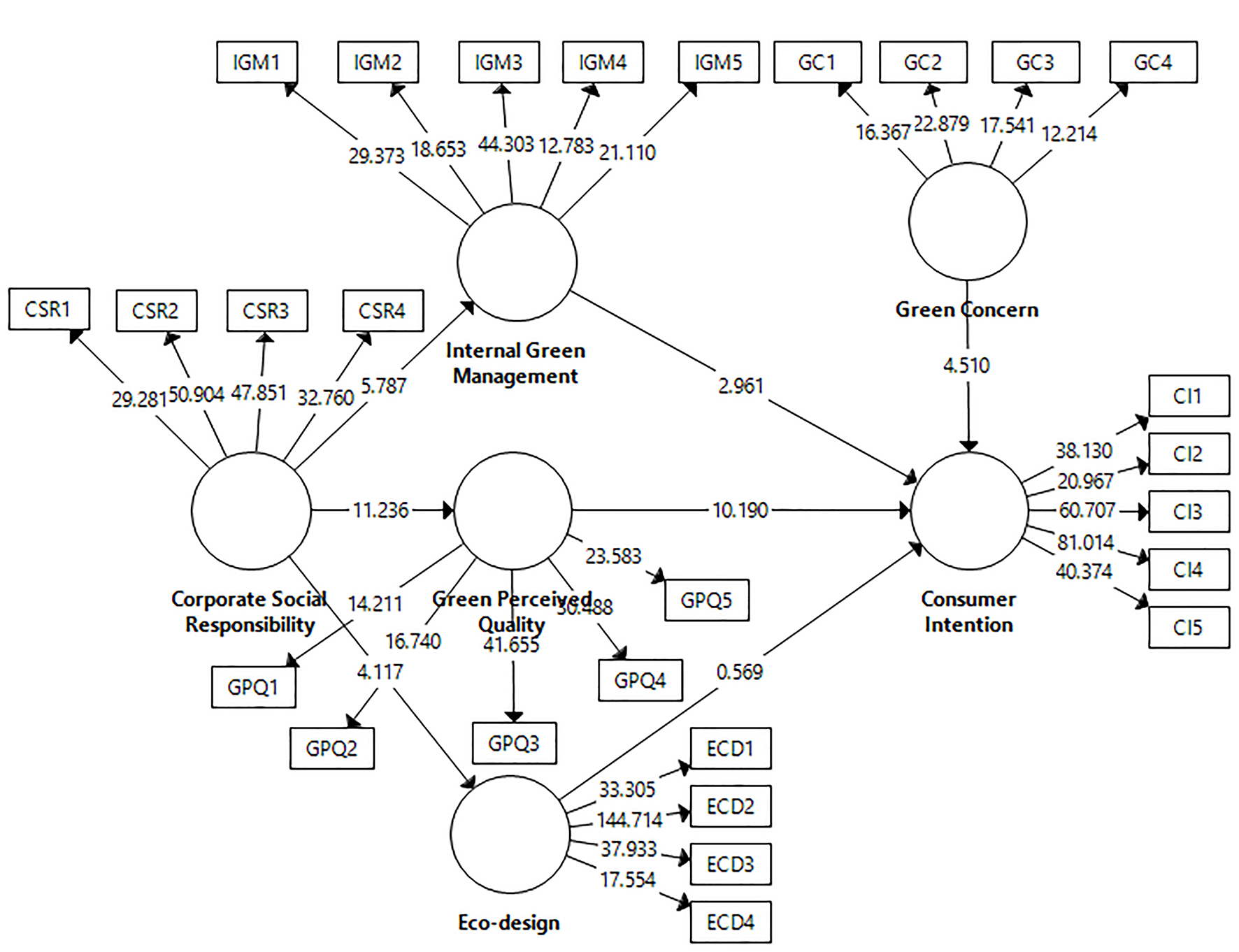

Supplement: Supplementary file 4 [file Image_1.PNG]
